# Australian School Staff and Allied Health Professional Perspectives of Mental Health Literacy in Schools: a Mixed Methods Study

**DOI:** 10.1007/s10648-023-09725-5

**Published:** 2023-01-21

**Authors:** Alexandra Marinucci, Christine Grové, Kelly-Ann Allen

**Affiliations:** Faculty of Education, Monash University, 19 Ancora Imparo Way, Clayton, VIC 3800 Australia

**Keywords:** Mental health literacy, School staff, Allied health professionals, Perspectives, School, Youth

## Abstract

Schools are an ideal setting to base mental health literacy programs as the learning environment is already established. Mental health literacy represents how to obtain and maintain good mental health and should be incorporated into the school curriculum. Collaboration with key stakeholders in youth mental health, such as school staff and allied health professionals, is critical in ensuring needs are addressed, and programs are sustainable. This study aimed to understand the perspectives of school staff and allied health professionals towards mental health literacy within Australian schools. An exploratory qualitative and quantitative survey was completed by 88 participants across Australia. Results indicated that school staff feel less competent to deliver mental health literacy content and perceive their training did not address mental health education compared to allied health professionals. Most mental health programs reported to be implemented within schools were social and emotional learning programs. School staff viewed mental health programs to strengthen academic performance less than allied health professionals. Thematic analysis of qualitative data identified that participants viewed mental health knowledge, where and how to seek help, and coping skills as vital content to cover in a school-based mental health literacy program. This study reveals that school staff need sufficient training and resources to address youth mental health literacy. Though mental health literacy is lacking in the Australian school system, the perspectives of school staff and allied health professionals in this study provide key ideas and considerations for the future development of school-based mental health literacy programs for youth.

**Supplementary Information:**

The online version contains supplementary material available at 10.1007/s10648-023-09725-5.

## Introduction

Mental health literacy is the knowledge of mental health, including symptoms of mental illness and positive mental health actions, how to seek help and support, and how to maintain one’s mental health (Bale et al., 2020; Jorm, 2012, 2020; Jorm et al., 1997; Kutcher et al., 2016). The World Health Organization (WHO, 2002) has claimed that the role of the school staff (e.g., teachers, well-being leaders, principals) is to cultivate student learning, including mental health literacy. Primary and secondary schools have been identified as an optimal setting for mental health promotion and prevention as there is access to youth, the learning environment is established, and most schools have existing programs and approaches that support the health, well-being, and social and emotional development of students (Conley & Durlak, 2017; WHO, 2005). Article 29 of the United Nations Convention on the Rights of the Child (1989) states that education includes the development of personality, physical ability, and mental health. The goal of education has moved away from a focus on academic performance only towards including the social, emotional, and mental health needs of youth (Cefai et al., 2021). This study focuses on the perspectives and experiences of Australian school staff and health professionals towards meeting the mental health educational needs of young people.

As part of meeting the needs of young people, the Australian National Children’s Mental Health and Wellbeing Strategy uses a well-being continuum to describe child mental health and well-being, ranging from healthy, coping, and struggling, to being mentally unwell (National Mental Health Commission, 2021). Key objectives of the Australian National Children’s Mental Health and Wellbeing Strategy suggest that schools provide a culture of well-being promoting mental health, adequate professional support, and use evidence-based mental health programs. A core objective of this strategy is to increase mental health literacy (MHL) for parents, carers, communities, and children through initiatives that are relevant to all populations (National Mental Health Commission, 2021). Vision 2030 provides a systematic plan for mental health prevention and highlights a need to co-design programs with the community to foster a continuum of care across age groups (National Mental Health Commission, 2022). Though these strategies have been put forward, little research exists to determine whether objectives are being met and whether school-based well-being and mental health programs are effective (Dix et al., 2020). There is a significant need to understand the perspectives of MHL of individuals who work with children and young people, such as school staff and health professionals. Collaboration and engagement are critical for ensuring the relevance, sustainability, and feasibility of proposed strategies to address needs (McDaid et al., 2017); however, little research has examined the perspectives of school staff and allied health professionals towards school-based MHL programs (Ekornes, 2015; Samnøy et al., 2020).

One in seven Australian children and adolescents have experienced a mental disorder (Lawrence et al., 2015), and Australian youth aged 12 to 25 years old have the lowest service access rate of any age group (Islam et al., 2020; McGorry et al., 2013). Since the introduction of the Better Access Scheme and headspace in Australia, access to services has increased; however, the prevalence of mental illness has also increased, suggesting that care in times of crisis is not enough and preventative approaches are needed (Jorm & Kitchener, 2020). The proportion of young Australians experiencing psychological distress has increased in the past eight years from 18.6 to 26.6% (Brennan et al., 2021). Youth report barriers to seeking help for mental health difficulties include both negative attitudes towards mental illness and seeking help, and poor knowledge of mental health and the help available (Marinucci et al., 2022b; Radez et al., 2020). Increasing the mental health literacy of youth could provide useful resources and skills that may decrease the prevalence of mental illness in the future (Jorm, 2019). School staff are an integral part of fostering positive youth development; therefore, their engagement and involvement in incorporating MHL into schools are essential (Marinucci et al., 2022a).

Involving school staff in the development, implementation, and evaluation of MHL programs could increase the probability of uptake and acceptance of MHL within Australian school settings in the future (Berzin et al., 2011; Feinstein et al., 2009). Research that has explored school staff perspectives of MHL and well-being in the curriculum highlights that school staff feel incompetent to deliver such information (Graham et al., 2011; Samnøy et al., 2020; Whitley et al., 2013). One study found that although a majority of their sample of Australian school staff were willing to be involved in MHL programs, less than half felt confident implementing MHL programs (Graham et al., 2011). Despite the WHO’s (2018) definition of mental health as more than the absence of illness, Australian primary and secondary school staff perceive mental health as pathology-oriented (Graham et al., 2011). There is a consensus on the importance of preventative approaches among school counsellors and school staff; however, school counsellors noted that their role was mainly reacting to crises and responding to the needs of high-risk students (Beames et al., 2020). School staff and counsellors also emphasized the importance of MHL for students and empowering students to seek help when necessary (Beames et al., 2020). A separate qualitative study reported school staff want advice from psychologists and need their presence in the school environment for support (Ekornes, 2015). There were clear concerns from school staff on the lack of access to mental health professionals in schools, and feeling inexperienced in identifying warning signs and the students’ need for professional help (Ekornes, 2015). Enhancing the MHL of school staff and students could significantly improve school staff competence and student well-being, with a critical shift in focus from reacting to crises to utilizing preventative approaches to mental illness (Kutcher et al., 2016). Whilst previous research highlights a need for support for school staff in supporting youth mental health (Ekornes, 2015; Mazzer & Rickwood, 2015), evidence is lacking in how school staff believe MHL could be enhanced in the school environment (Beames et al., 2020). Given school staff are with young people often, their perspectives are crucial (World Health Organization, 2002).

A Norwegian study found that school staff want to be informed and supported by mental health professionals in the classroom (e.g., a psychologist or counsellor) and to work as a collaborative team (Mælan et al., 2020). During the COVID-19 pandemic, school psychologists in Australia reported an increase in daily working hours and providing universal social-emotional and mental health support (Reupert et al., 2022). The Australian Psychological Society recommends a ratio of one psychologist to 500 students in a school setting (Australian Psychological Society, 2016). Currently, 10.8% of registered Australian psychologists work in schools (Australian Institute of Health Welfare, 2021); however, this is not enough to meet the current needs for mental health services (Australian Psychological Society, 2022). If available, young people receive support from counsellors or psychologists through school (Hall et al., 2019). The effects of the COVID-19 pandemic on mental health and the health system have led to an increased need for services. Barriers to accessing support include long wait times for services, an overworked and limited workforce, and financial costs of services (Byrne et al., 2021; Serafini et al., 2020; Singh et al., 2020). During the COVID-19 pandemic, school psychologists in Australia reported an increase in daily working hours and providing universal social-emotional and mental health support (Reupert et al., 2022). School psychologists provide a wide range of services, including:

- Assessment and evaluation for educational and developmental difficulties;
- Supporting parents, teachers, and communities;
- Consulting on school-wide interventions;
- Implementing prevention and intervention programs to develop well-being;
- Facilitating individual or group work for specific needs (American Psychological Association, 2022; National Association of School Psychologists, 2021).

MHL could be systemically incorporated into the curriculum by school staff to support the mental health of young people to increase help-seeking actions, coping strategies, and knowledge of mental health (Jorm, 2012; Weare, 2017). Mental health professionals or psychologists could be employed in schools to deliver and support school-based mental health programs to allow for a holistic approach to MHL for students. This could be further enhanced by providing training to school staff to support school-based mental health programs.

The literature recommends that collaboration with key stakeholders be initiated during the development of programs to ensure appropriate needs are addressed and research is guided by the target population such as students and school staff (Cowie et al., 2004; World Health Organization, 2005). It is unclear whether school-based MHL programs in the literature have been developed based on school staff and allied health professional contribution and guided by the needs of schools (Marinucci et al., 2022a; Ojio et al., 2019; Painter et al., 2017; Patalay et al., 2017; Perry et al., 2014). These contributions are important not only for the content of programs, but also for sustainability and feasibility if programs were to be implemented long-term at a wide scale (Hagermoser Sanetti & Collier-Meek, 2019; Santor & Bagnell, 2012). Specifically, within an Australian context, little research has been conducted to understand the views of those directly involved with enhancing youth mental health, including what should be integrated into schools with the direct purpose to increase youth MHL.

This study aims to understand the perspectives of Australian school staff and allied health professionals on MHL in school settings and their views of an example MHL program. This study is guided by the following research questions:

1. What are the perspectives and experiences of school staff and allied health professionals towards mental health education within an Australian school setting?
2. What are school staff and allied health professionals’ perspectives on school-based mental health literacy programs?
3. Do the perspectives differ between school staff and allied health professionals towards school-based mental health education and mental health literacy programs?
4. What school-based mental health programs are currently used across Australian schools?

School staff include well-being leaders and school teachers, and allied health professionals include psychologists, counsellors, art therapists, speech pathologists, and mental health clinicians. It is anticipated this study will contribute to evidence supporting the inclusion of key stakeholders, (such as school staff and allied health professionals) in mental health education research. This will highlight the need for collaboration during the development and implementation of MHL programs.

## Method

### Research Design

This mixed-methods study used a survey to obtain data to gain insight into the perspectives of school staff and allied health professionals. Though qualitative data are traditionally collected through interviews, evidence shows that qualitative surveys can provide focused data in an unobtrusive manner (Braun et al., 2020). The study was approved by the relevant University Human Research Ethics Committee (Project ID: 27638). An explanatory statement was provided, and informed consent was implied when participants completed the anonymous survey. Given this is an exploratory study to understand the views of school staff and allied health professionals, a mixed-methods approach was used to both quantify findings and gather in-depth qualitative data. This approach was used to integrate diverse methods and develop a comprehensive understanding of perspectives (Plano Clark, 2019). Quantitative data were obtained to understand participant experiences generally and quantify information and were analyzed using descriptive statistics, Welch’s *t*-tests, and frequency analysis. Qualitative data were gathered to understand participant perspectives within their individual context and towards an example MHL program and analyzed using deductive thematic analysis.

### Participants

Participants were recruited for the study using snowballing via social media advertisements and posts from the researchers. Participants were informed of the survey’s focus prior to consenting to participate. The survey data were collected from April to July 2021 across all areas of Australia. Inclusion criteria included school staff and allied health professionals who worked in a school environment. A total of 159 responses were recorded using Qualtrics (2021); however, 53 responses were excluded due to blank entries (*n* = 44), missing professional role (*n* = 5), or excluded populations (*n* = 4). Participants with more than 20% of missing data were removed from the sample (*n* = 18). After data cleaning, the total sample size was 88 participants. Participants who identified as health professionals were an art therapist (*n* = 1, educational background [EB] = postgraduate degree), mental health clinician (*n* = 1, EB = postgraduate degree), provisional psychologist (*n* = 1, EB = bachelor degree), psychologist (*n* = 3, EB = postgraduate degree), or speech pathologist (*n* = 2, EB = bachelor degree/postgraduate degree). Participant characteristics are outlined in Table 1. For participants that stated “other” for the type of school, these were allied health professionals (*n* = 8) and school staff (*n* = 5) who worked across multiple types of schools or worked in early years to year 10 or 12 schools.

**Table 1:** Participant characteristics (*N* = 88)

Category	Sub-category	*n* (proportion %)
Gender	Male	4 (4.5%)
	Female	81 (92.1%)
	Intersex, trans, gender fluid, or gender diverse	1 (1.1%)
	Not provided	2 (2.3%)
Age (years)	18–24	6 (6.8%)
	25–34	19 (21.6%)
	35–44	15 (17.1%)
	45–54	36 (40.9%)
	55–64	12 (13.6%)
Educational background	High school certificate or equivalent	1 (1.1%)
	Vocational qualification	2 (2.3%)
	Associate qualification	1 (1.1%)
	Bachelor degree	23 (26.1%)
	Postgraduate degree	60 (68.2%)
	Other	1 (1.1%)
State	Australian capital territory	2 (2.3%)
	New South Wales	10 (11.4%)
	Queensland	16 (18.2%)
	South Australia	8 (9.1%)
	Tasmania	2 (2.3%)
	Victoria	39 (44.3%)
	Western Australia	11 (12.5%)
Professional role	School teacher	47 (53.4%)
	School psychologist/counsellor	28 (31.8%)
	School well-being leader	5 (5.7%)
	Health professional	8 (9.1%)
Type of school	Primary	36 (40.9%)
	Secondary	32 (36.4%)
	Both	7 (8.0%)
	Other	13 (14.7%)
Years in profession	0–5	29 (33.0%)
	6–10	8 (9.1%)
	11–15	16 (18.2%)
	16–20	8 (9.1%)
	21–25	13 (14.8%)
	26+	14 (15.9%)

### Data Analysis

Analysis was conducted using the Statistical Package for the Social Sciences (SPSS), version 27.0.1.0 (IBM, 2021). Quantitative data from the survey were analyzed using descriptive statistics, Welch’s *t*-tests, and frequency analysis. School teachers and well-being leaders were grouped and recorded as *school staff* (*n* = 52). School psychologists/counsellors and health professionals were grouped together and recorded as *allied health professionals* (*n* = 36).

Frequency analysis was used for quantitative items. Welch’s independent samples *t*-tests were conducted to identify mean differences between groups across quantitative Likert-scale items. This method was chosen to reduce type I error rates as the assumption of homogeneity of variance was violated. Hedge’s g was used to provide the corrected effect size.

Qualitative data from the survey for general questions (e.g., “What is covered in health education associated with mental health? Is it helpful? Why/Why not?”) and specific questions for the example MHL program (e.g., “Are there any areas of mental health education that are missing from the [example MHL] program?”) were extracted and analyzed according to Braun and Clarke’s (2006, 2012, 2021) six-phase approach for thematic analysis. A deductive approach was taken, as questions were specific and predetermined. One author developed the coding frame and coded the data. For validity and inter-rater reliability of the analysis, two authors reviewed the coding process (O’Connor & Joffe, 2020). The three authors negotiated to come to a consensus on the interpretation of results and ensure the trustworthiness of the analysis (Cheung & Tai, 2021). The themes generated were as follows: (1) basic mental health education in the current curriculum, (2) content to be included in MHL programs, and (3) suggestions for future MHL programs. Themes were analyzed according to the groups of school staff and allied health professionals. As the first theme (basic mental health education in the current curriculum) aligns with general perspectives of current mental health education, this has been presented accordingly in the results section

Analysis of reported school-based mental health programs reported by participants was classified into social and emotional learning, mental health literacy, or well-being programs and evaluated for whether they were evidence-based by identifying literature or reports evaluating the program and consulting the systematic review by Dix et al. (2020).

### Development and Design of the Survey

A 51-item quantitative and qualitative survey (not including demographics) was used to collect data (see Supplementary Information Table S.1 for the full survey). This was created based on previous research examining school staffs’ perspectives and covered topics identified through a review of the literature and recommendations from researchers in the field of educational and developmental psychology (Askell-Williams & Cefai, 2014; Beames et al., 2020; Moon et al., 2017; Russet et al., 2022). Informed by the literature, the relevance and clarity of each survey item were developed, reviewed, and negotiated by the three authors to ensure items measured what they intended to measure. The individual items in the survey do not collectively create a reliability-tested index or scale but rather provide descriptive data on respondents’ perspectives of mental health literacy in Australian schools (Braun et al., 2020). The development of the survey was qualitative in nature, as this study was an exploratory study.

Thirty quantitative items made up the first part of the survey. Quantitative statement items were rated using a 5-point Likert scale to understand general perspectives towards mental health literacy and mental health education in the school environment. Ratings depended on the item, for example: strongly disagree, somewhat disagree, neither agree nor disagree, somewhat agree, strongly agree; extremely useful, very useful, moderately useful, slightly useful, not at all useful; extremely important, very important, moderately important, slightly important, not at all important. An example quantitative item was, “I feel competent delivering mental health education to students.”

The second part of the survey included 15 qualitative items with an open unlimited response. Qualitative data were gathered using text entry questions to gain rich insight into the participants’ views (Braun & Clarke, 2014). General questions were asked to gather information about their current knowledge of the school health curriculum and the use of mental health programs. An example qualitative item was, “What school-based mental health programs, if any, have been implemented in your school?”. Details of an example mental MHL program were provided, such as session structure and learning objectives, and specific questions were asked to identify their professional views of the program. The example MHL program was the Australian adapted Youth Education and Support (YES) program. The YES program runs for ten sessions over 10 weeks, and participants were asked what they liked, did not like, and what they would change about sessions, see Table 2 for the program overview (Marinucci et al., 2021). An example qualitative item about the YES program was:

**Table 2:** Overview of the Australian adapted YES Program from Marinucci et al. (2021)

Session	Topic/s
1. Introduction	Expectations of the program, physical signs of stress
2. Coping	Positive and healthy coping behaviors
3. Mental illness and recovery	Defining mental illness, recovery, and prevalence
4. Depression and anxiety	Depression and anxiety symptoms, managing symptoms
5. Coping and resilience	Individual coping strategies
6. Help seeking and support	Where and how to seek help and help others
7. Stigma	Mental illness stigma
8. Families	Mental illness in families
9. Remember and hope	Values and goals, reviewing content
10. Graduation	Reflection and graduation from the program

Week 4: depression and anxiety

- Understand emotions and behavior associated with depression
- Learn some strategies to manage depressive and/or anxious symptoms

Week 5: coping and resilience

- Develop individual coping strategies
- Understand healthy behaviors to promote resilience and coping
- What, if anything, do you like about weeks 4 and 5 of the YES program?

## Results

This study explored the perspectives of school staff and allied health professionals towards mental health education and mental health literacy. The data are reported in four sections, with each section addressing one or two of the research questions.

### General Perspectives of Current Mental Health Education

**Fig. 1:**
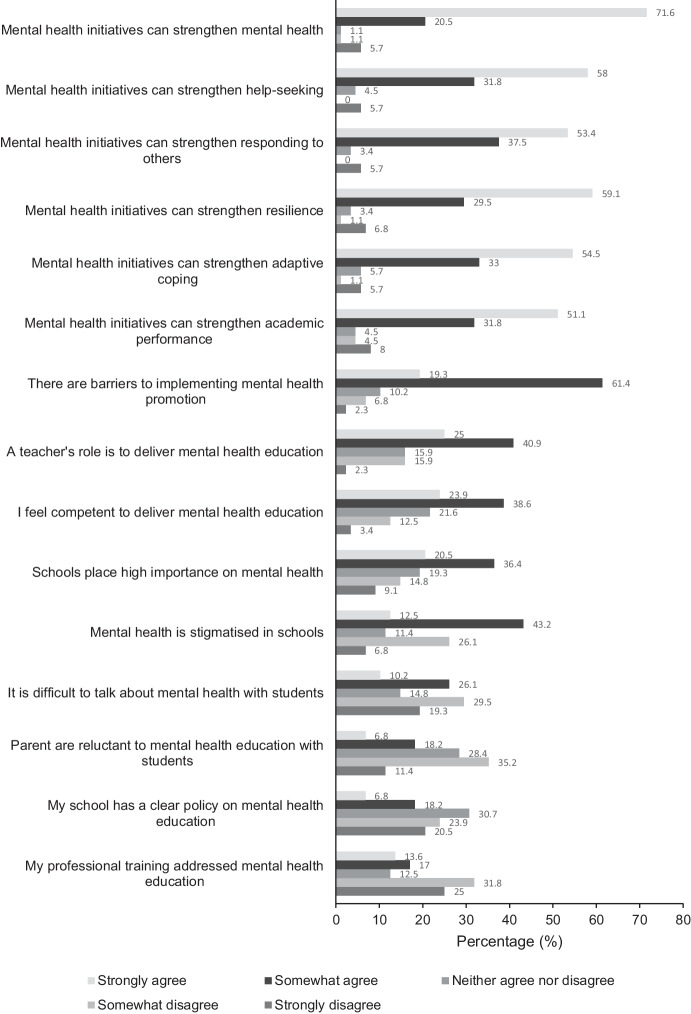
Frequency of responses of the overall sample (*n* = 88) for items rated strongly disagree (1) to strongly agree (5)

**Fig. 2:**
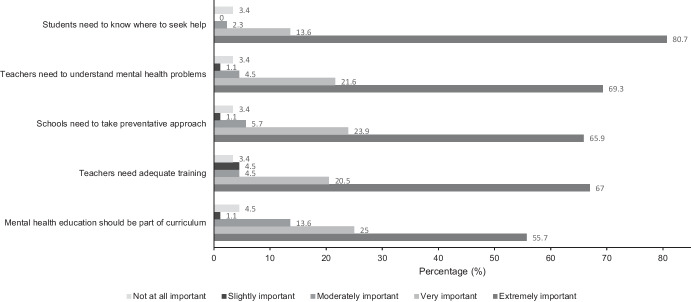
Frequency of responses of the overall sample (*n* = 88) for items rated extremely important (1) to not at all important (5)

**Fig. 3:**
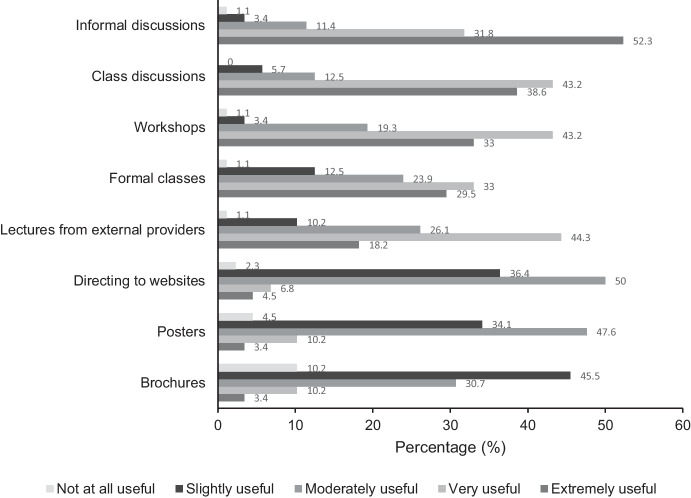
Frequency of responses of the overall sample (*n* = 88) for items rated extremely useful (1) to not at all useful (5)

The following results address the first and third research questions: (1) What are the perspectives and experiences of school staff and allied health professionals towards mental health education within an Australian school setting? and (2) Do the perspectives differ between school staff and allied health professionals towards school-based mental health education and mental health literacy programs? For the overall sample, a high proportion of participants strongly agreed that mental health initiatives could strengthen mental health, help-seeking behavior, responding to others, resilience, adaptive coping, and academic performance. School staff somewhat agreed (30.8%) that it is difficult to talk about mental health with students; however, allied health professionals somewhat disagreed (33.3%) with this statement. School staff strongly disagreed (26.9%) that their school had a clear policy on mental health education, whilst allied health professionals neither agreed nor disagreed (38.9%). See Figs. 1, 2, and 3 for the distribution of responses, and see Table S2, Table S3, and Table S4 in the Supplementary Information for mean and standard deviation scores.

Many participants responded that the following are extremely important: addressing the need for students to know where to seek help (*n =*71), teachers to have adequate training (*n* = 59) and understand mental health problems (*n* = 61), schools to take a preventative approach (*n* = 58), and mental health education to be part of the curriculum (*n* = 49). The distribution of responses did not differ for school staff and allied health professionals. Informal discussions were perceived as the most useful resource for mental health education and this did not differ for school staff (53.8%) and allied health professionals (52.3%).

Welch’s *t-*test found significant differences in “My professional training addressed mental health education for students” between school staff (*M* = 1.96, *SD* = 1.07) and allied health professionals (*M* = 3.58, *SD* = 1.23; *t* (68.29) = −6.43, *p* < .001), and the magnitude of the difference was large (Hedge’s *g* = 1.42). The item “I feel competent delivering mental health education to students” was significantly different for school staff (*M* = 3.27, *SD* = 1.09) and allied health professionals (*M* = 4.25, *SD* = .77; *t* (85.94) = −4.96, *p* < .001, *g* = 1.00). On the item “School based mental health initiatives can strengthen academic performance”, school staff (*M* = 3.87, *SD* = 1.37) agreed less so than allied health professionals (*M* = 4.53, *SD* = .77; *t* (83.08) = −2.88, *p* = .005, *g* = .56). No significant mean differences were found for participants who worked in a primary school compared to a secondary school.

The first theme generated from the deductive thematic analysis of the qualitative survey data encompassed a general view that mental health education was taught at a superficial level within the school environment. There were varied responses to the level of mental health content covered in the health education curriculum. Some school staff participants reported they did not teach health and therefore did not know what was taught. Others reported little mental health content was covered: “Very basic information not linked to young people’s modern experiences” (school teacher), or the mental health content was not sufficient: “No as [mental health education is] very surface level and many teachers don’t feel they have the capacity to teach delicate topics” (school teacher), and “Too often…there can be a message given that if you do X, Y and Z that you will not be a person with mental health conditions and if you don’t do these things you are not showing resilience…more education about stigma is needed” (school teacher). School staff reported a reluctance to teach mental health content: “Teachers should not have to take this extra load on” (school teacher). This was attributed to a lack of training in the area of mental health and a lack of time to cover such content in an already busy curriculum: “It’s a matter of finding the time to fit things in on top of all the curriculum priorities” (school teacher), and “We [teachers] are not trained and we already have far to [sic] much to teach…the legal remifications [sic] of teaching lead me to wonder if we are to teach something and it is not understood and something happens, then we are to blame” (school teacher).

Allied health professionals were unsure of what was taught: “I’m not actually sure. The psychologists aren’t involved sadly” (school psychologist/counsellor), or reported an observed reluctance from teachers: “Teachers are too afraid, and not trained suitably, to deliver mental health content” (school psychologist/counsellor). Overall, allied health professionals stated that basic mental health education was covered; however, this was not sufficient for students.

Overall, participants noted that resilience, acceptance of differences, emotional awareness, growth mindset, mindfulness, coping strategies, relaxation techniques, or help-seeking strategies were covered in the school curriculum. Information about mental illnesses, such as anxiety, depression, and eating disorders, was reported to be covered in the health curriculum by 12 participants. One allied health professional participant (speech pathologist) reported that resilience and stress management were covered but specifically framed in the context of academic and study skills in the school environment. Two participants reported “vaping, sexting, bullying, respectful relationships” (school psychologist/counsellor) and “sex ed[ucation]” (school teacher) were covered as mental health education.

### Perspectives of Roles in the School Environment to Support Student Mental Health

**Fig. 4:**
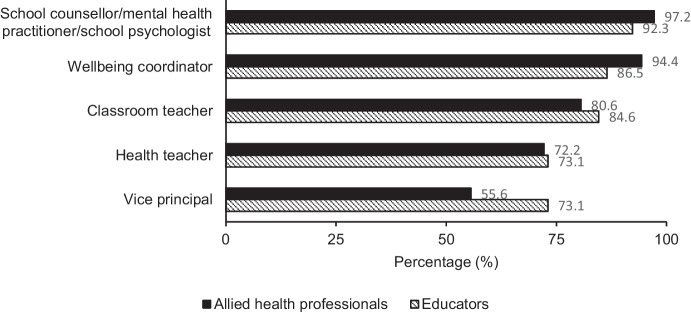
Frequency of responses for who should deliver mental health education by school staff (*n* = 52) and allied health professionals (*n* = 36)

These results address the first research question: What are the perspectives and experiences of school staff and allied health professionals towards mental health education within an Australian school setting? Most participants agreed that the role of the school well-being team and teachers includes supporting and fostering positive mental health in students. A high percentage of participants selected school counsellors/mental health practitioners/school psychologists, well-being coordinators, and classroom teachers as professionals most suited to delivering mental health education, with a lower percentage selecting health teachers and vice principals. Figure 4 displays the spread of responses of school staff and allied health professionals for the most suitable professionals to implement mental health education.

**Fig. 5:**
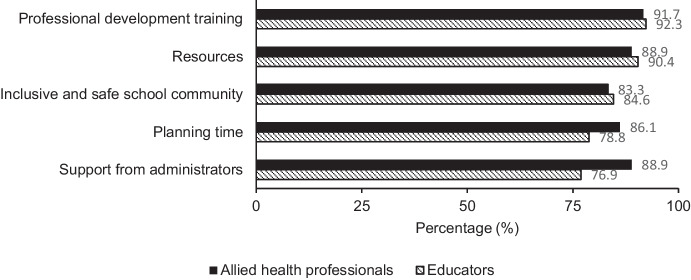
Frequency of responses for resources required by school staff (*n* = 52) and allied health professionals (*n* = 36)

For mental health education to be included in the curriculum, participants reported they would need professional development training, resources such as teaching materials, an inclusive and safe school community, planning time, and support from administrators. Participants were able to provide additional details of what would be needed (*n* = 17) and reported that a health professional would be needed to deliver the content and support students, funding would be required, more time or other subjects were taken out from the curriculum, parental support given, and additional trained staff. Figure 5 displays the spread of responses from school staff and allied health professionals for resources required for mental health education to be included in the curriculum.

### Perspectives Towards Mental Health Literacy

This topic addresses the second and third research questions: (2) What are the perspectives of school-based mental health literacy programs for school staff and allied health professionals? and (3) Do the perspectives on school-based mental health education and mental health literacy programs differ between school staff and allied health professionals? The second theme generated from the deductive thematic analysis of qualitative data suggested content participants agreed should be included in MHL programs. Table 3 presents the content viewed as desirable to include in MHL programs reported by school staff, and allied health professionals, and shared across both groups.

**Table 3:** Desirable content for mental health literacy programs

	School staff	Allied health professionals	Both groups
Introducing the program to students	✓		
Increasing mental health vocabulary	✓		
Addressing stigma of mental health	✓		
Increasing emotional awareness		✓	
Normalizing mental health		✓	
Understanding heritability of mental illness		✓	
Mental health within families			✓
Recognizing and identifying signs of mental health challenges			✓
Setting goals based on personal values			✓
Help-seeking—formal and informal sources of support, how to seek help			✓
Creating a coping plan (i.e., personal signs of stress, people to talk to, actions to take)			✓
Increasing awareness of mental health and coping skills			✓
Explicitly teaching students about mental health			✓
Understanding the process of therapy			✓

✓, reported by group

The final theme explored suggestions and considerations from the participants for future MHL programs. Overall, there is some overlap in suggestions between school staff and allied health professionals, as presented in Table 4.

**Table 4:** Suggestions and considerations for future mental health literacy programs

School staff	Allied health professionals
**•** Collaborate with students through feedback in programs **•** To have individual MHL programs for students **•** Practicality of programs considered during development **•** Ensure there is interactivity such as video clips **•** Focus on idea that mental health is valuable for everyone and have a large focus on help seeking **•** Problem solving strategies to manage mental illness symptoms **•** Delivered by trained health professionals and follow ups included for ongoing support, including ongoing learning to target stigma **•** Include case studies and target how to identify mental illness in others, lived experiences of mental illness, effects of bullying, suicide, and mental health first aid **•** Have family involvement and homework activities to create discussions at home **•** MHL programs integrated in to daily school life through a whole school approach **•** Teaching students how to avoid damaging sources of information **•** Including support for at-risk students such as a mental health professional present to answer complex questions **•** Resources for teachers and training for staff **•** Conversations are sensitive to neurodivergence and mental health	**•** Ensuring safety for students who may feel triggered by content **•** Multimodal delivery of programs **•** Teaching students about different types of therapies, including non-talk therapies **•** Discussion around mental wellness and a healthy living plan including diet, sleep, socializing, religion, faith, spirituality, and healthy boundaries **•** Programs need to be contextually appropriate, with cultural factors and living arrangements considered (e.g., children in out of home care) **•** Using the biosocial model or biopsychosocial model to explore factors of prevalence and heritability of mental illness **•** Inclusion of maintaining factors in mental health, the connection of thoughts, feelings and behavior, and identification of unhelpful thinking styles **•** Have less emphasis on the disorder, illness, and clinical presentations in relation to mental health **•** Guest speakers for the lived experience of mental illness **•** Use practical skills for coping strategies **•** Differentiate between stress and anxiety for students **•** Explain what well-being looks like, normalize all emotions and focus on strengths **•** Include case studies, the mental health continuum, gender issues, bullying prevention, procrastination, test anxiety **•** Build a healthy and safe environment in the school **•** How to manage stress broadly, and how to help and support friends and family **•** Creating boundaries and teaching students to go to a trusted adult **•** Consider the impact on students where there is severe family mental illness, and have explicit conversations if a parent’s mental ill health is impacting them

School staff were concerned with fitting a MHL program in an already busy curriculum and making it suitable for students across all ages. School staff reported that students may not engage with information about mental illness if they have not experienced mental illness or if they have limited emotional understanding. An important limitation of available help was raised, as resources may be limited across different geographical and socio-demographic areas: “I feel that a lot of schools are under-resourced, and we could be setting them up to fail as they do not have the resources to follow up with those students who require support” (school teacher). A holistic approach to mental health across the whole school was reported as needed, specifically in rural, remote, and regional schools. It was noted that mental health should be framed positively and that discussing mental health could impart to students that they should or will struggle with mental illness: “Are we inserting these views into primary school children before they are ready?” (school teacher) and “What is the program doing for adolescents who are not struggling with mental health? Are we suggesting to them that they should be?” (school teacher). Discussing family mental health and heritability was viewed as a loaded topic that could be difficult to include in a program suitable to a wide range of students, such as students in out-of-home care. One school staff mentioned that covering content related to mental health stigma could be difficult for some teachers: “It challenges teachers with their own attitudes to stigma” (school teacher). Lastly, the need for school staff support and training and medical professionals liaising with schools was suggested to implement into an MHL program.

Allied health professionals held similar views to school staff related to discussing familial mental illness and heritability: “Not sure I like the focus on families. What does this teach them? Could have negative implications about their own family” (school psychologist/counsellor) and “Heritability can be misinterpreted by some students as a given that they will develop mental illness if their parent is not well” (school psychologist/counsellor). There was reluctance to discuss stigma, with concern that this could reinforce negative attitudes: “Why reinforce stigma with the use of this term, it continues to entrench a deficient mindset” (art therapist). Participants noted that a range of mental illnesses should be discussed: “You can’t just talk about anxiety and depression. These may be the focus, but it would be damaging…not to talk about psychosis” (school psychologist/counsellor). On the other hand, it was requested for programs to focus less on clinical diagnoses: “Focus on depression and anxiety – could it be less clinical?” (school psychologist/counsellor). Allied health professionals reported programs would be limited by time constraints in the school environment and the student’s ability to self-reflect.

### School-Based Programs Analysis

The following data aimed to address the fourth research question: What school-based mental health programs are currently used across Australian schools? Participants reported that various school-based programs were implemented in their schools. These programs were categorized according to targeted areas of social and emotional learning, mental health literacy, and well-being. Programs were determined as evidence-based through identifying literature evaluating the program and through consulting the systematic review from Dix et al. (2020). Table 5 presents each program with its associated category, how many participants reported using it, and the presence of evidence with an example citation.

**Table 5:** School-based programs reported by participants

	Type	Evidence-based?	Number of participants	Example citation
Aussie Optimism	SEL	Y	1	Myles-Pallister et al. (2014)
batyr@school	MHL	Y	1	Hudson and Ingram (2017)
BITE BACK	WB	Y	1	Manicavasagar et al. (2014)
Bounce Back!	SEL	Y	2	Noble and McGrath (2018)
Feeling Fantastic	WB	N	1	None available
Flourish Girls	SEL	N	1	None available
Friendly Schools Plus	SEL ^b^	Y	1	Cross et al. (2018)
Friends for Life	SEL	Y	1	Briesch et al. (2010)
Journey of Hope	SEL	Y	1	Powell and Bui (2016)
Leader in Me	SEL	Y	2	Soutter (2018)
ManCave	SEL	Y	1	The Man Cave (2021)
MindMatters	SEL	Y	2	Wyn et al. (2000)
MindUP	SEL	Y	1	Schonert-Reichl et al. (2015)
PATHS Curriculum	SEL	Y	2	Humphrey et al. (2016)
Pause Program	WB	N	1	None available
Protective Behaviors	WB ^a^	Y	1	Rose (2004)
Resilience, Rights and Respectful Relationships	SEL	Y	3	Kearney et al. (2016)
Rock and Water	SEL	Y	1	Ykema et al. (2006)
Seasons for Growth	WB	Y	2	Frydenberg et al. (2006)
Teen Mental Health First Aid	MHL	Y	4	Hart et al. (2016)
The Best of Coping	SEL	Y	1	(Frydenberg et al., 2004)
The Girl Campaign	WB	N	1	None available
The Resilience Project	SEL	Y	8	The Resilience Project (2021)
What’s the Buzz?	SEL	N	2	None available
You Can Do It! Education: Program Achieve	SEL	Y	4	Ashdown and Bernard (2012)
Youth Aware of Mental Health	MHL	Y	1	Lindow et al. (2020)
Zones of Regulation	SEL	Y	7	Nowell et al. (2019)

*Note. MHL*, mental health literacy; *SEL*, social and emotional learning; *WB*, well-being

^a^This program focuses on personal safety

^b^This program also focuses on bullying

It was also reported that various frameworks were used in school, such as the School-Wide Positive Behavior Support (Department of Education, 2021b), the Discover, Notice, Advisor and Values (DNA-V) model of thriving (Ciarrochi et al., 2016), the Berry Street Education Model (2021), and the Positive Behavior for Learning (Department of Education, 2021a). The school staff professional learning training, SAFEMinds (headspace, 2021), was offered at one school. Participants also reported they used websites with various resources included Smiling Mind (2021), Wheel of Well-Being (2013), and BeYou (Beyond Blue, 2021). Programs from organizations were referred to, such as the Black Dog Institute (2021), Educator Impact (2018), and yourtown (2021), though specific programs were not provided. The program Rumble’s Quest (RealWell, 2021) was reported to be used by one participant; however, this is not a school-based program. Cognizance (Independent Schools Victoria, 2021), a program focusing on metacognition, R.A.G.E (Interventions Plus, 2017), an anger management program, and Love Bites (NAPCAN, 2021), a domestic and family violence and sexual assault prevention program, were reported to be implemented. Some participants did not state a specific program; however, they reported the presence of chaplains, counsellors, or psychologists in the school environment that were accessible to students. Curriculum content covering social skills, social and emotional learning, and resilience were implemented. Ten participants reported no school-based mental health program has been implemented in their school. Overall, various school-based programs targeting social and emotional learning and aspects of mental health and well-being have been delivered within schools. It appears schools captured in this study focus on resilience, social skills, and emotion regulation through their mental health and well-being curriculum.

## Discussion

This study aimed to explore the perspectives and experiences of school staff and allied health professionals towards mental health education and school-based mental health literacy programs. Overall, school staff and allied health professionals perceived mental health to be somewhat stigmatized in the school environment, and school staff found it difficult to discuss mental health with students. This could be explained by student-teacher relationships that allow for discussions of mental health. A study based in Canada found that school staff (teachers, school counsellors, psychologists, administrators, support staff) viewed establishing and maintaining strong relationships with students as critical to addressing student mental health and stigma (Dimitropoulos et al., 2021). Stigma towards mental illness may be evident at the whole-school level, and previous research has highlighted some school staff do not believe it is their role to include mental health promotion in their curriculum (Askell-Williams & Cefai, 2014; Corcoran & Finney, 2015). The overall school climate has been linked with mental health stigma (Townsend et al., 2017); therefore, negative attitudes towards mental health held by school staff may contribute to stigma in the school environment. Participants reported that their school did not have a clear policy for teaching mental health, contributing to mental illness stigma.

In the current study, school staff and allied health professionals reported reluctance from teachers to deliver mental health education due to a lack of training, prioritizing the academic curriculum, and perceived legal ramifications. It was reported that if mental health education was covered, it was insufficient. Barriers to school-based mental health education, including pressure to focus on the academic curriculum, perceived inefficacy, and lack of adequate training are evident in the literature (Ekornes, 2015). Fear of legal ramifications for teaching mental health content may originate from a lack of confidence, as Mazzer and Rickwood (2015) found that Australian teachers reported fear of saying or doing the wrong thing and having concerns about being responsible for student mental health. Participants in this study viewed supporting student mental health as important and part of a teacher’s role; however, there remain barriers to including mental health education in the Australian curriculum.

Although all participants in the current study reported teachers and school well-being teams have a role in supporting student mental health, competency and professional training to deliver mental health education were significantly different among school staff and allied health professionals. Allied health professionals reported their professional training addressed mental health education and felt more competent to deliver MHL content than school staff. This is consistent with previous research demonstrating that whilst school staff perceive schools are responsible for student mental health (Beames et al., 2020; Moon et al., 2017; O’Reilly et al., 2018), competency is lacking due to limited resources and training (Ekornes, 2017; Mazzer & Rickwood, 2015; Reinke et al., 2011). Our sample of allied health professionals contained predominantly psychologists. In Australia, psychologists are required to complete undergraduate and postgraduate studies spanning approximately 5 to 6 years (Psychology Board of Australia, 2020). Therefore, it is expected that our sample of allied health professionals would feel more competent in delivering MHL content given their extended training in mental health. Schools have been recognized as an important setting to integrate mental health education and MHL (Hoare et al., 2020; O'Connor et al., 2018), though there is a clear need to upskill school staff who are in regular contact with students in youth mental health.

Aligned with previous research (Beames et al., 2020; Graham et al., 2011; Moon et al., 2017), for both school staff and allied health professionals, there was high importance placed upon preventative approaches, mental health education in the curriculum, and adequate training and understanding of youth mental health for professionals involved in student well-being. Participants recognized the need for students to know where to seek help, which is particularly important in supporting youth mental health (Radez et al., 2021). Overall, it was recognized that mental health education and MHL content could strengthen aspects of the student including resilience and help-seeking, although school staff perceived mental health education to strengthen academic performance less so than allied health professionals. This discrepancy could be due to a lack of knowledge of youth mental health among our sample of the school staff. Previous research indicates many Australian teachers hold a binary understanding of mental health that is pathology-oriented, rather than focusing on the strengths and capacities of the student (Graham et al., 2011). From this perspective, the link between academic performance and mental health may not be recognized.

Informal and class discussions were perceived as the most useful mode of mental health resource delivery, consistent with literature positing that facilitated discussions are effective for mental health promotion and development of MHL (McAllister et al., 2018). For programs targeting mental health within schools, most programs were focused on social and emotional learning (SEL) or overall well-being, rather than MHL. It is of concern that some programs implemented had no evidence base because evidence-based practice ensures quality care, continued professional development, and practice that is guided by appropriate research evidence (Kretlow & Blatz, 2011; Prasun, 2013). In Australia, SEL has become widely implemented in schools and is part of the curriculum (Australian Curriculum, 2021). SEL focuses on self-awareness, self-management, social awareness, relationship skills, and responsible decision-making (CASEL, 2021; Grové & Laletas, 2020), whereas MHL focuses on knowledge of mental health and how to obtain and maintain good mental health (Jorm, 2012; Kutcher et al., 2016). A recent study found that Australian school principals often implement mental health programs with little or no evidence of effectiveness, highlighting the need to support school staff in identifying, implementing, and evaluating evidence-based mental health programs (Laurens et al., 2021). It is evident that aspects of mental health are becoming part of the curriculum in Australian schools; however, research must be translated to practice through evidence-based programs targeting MHL in schools as a preventative approach towards the development of mental illness (Jorm, 2020; Marinucci et al., 2021).

Consulting and collaborating with key stakeholders, such as school staff and allied health professionals, is crucial when developing MHL programs to ensure feasibility and sustainability (McDaid et al., 2017; WHO, 2005). Involving school staff in addressing youth mental health is beneficial as they are with students often, understand the needs of the community, and are familiar with the school environment (Chesterson, 2009; WHO, 2002). The current study is the first to explore the perspectives of school staff and allied health professionals on content for MHL programs. School staff highlighted the importance of introducing the program to students, increasing mental health vocabulary, and addressing mental illness stigma. Whereas allied health professionals focused on normalizing mental health as an important aspect of MHL programs. School staff and allied health professionals viewed recognizing signs of mental illness, help-seeking, coping skills, and understanding therapy as content that needed to be covered in the school environment.

Suggestions for MHL programs varied between school staff and allied health professionals. School staff perceived discussing mental health content as triggering for some students; however, research on student perspectives identifies that increasing education and awareness of mental health are important to reduce stigma and negative attitudes (Chandra & Minkovitz, 2007; Moses, 2010). Allied health professionals and school staff perceived discussing family mental health and parental mental illness as a difficult topic that may not be relevant for all students. Approximately 21 to 23% of Australian children live in a family with parental mental illness (Maybery et al., 2009). Though this content may not be relevant to all students, children of parents with a mental illness (COPMI) want to receive information about mental illness and support at their school with their peers (Grové et al., 2016; Grové et al., 2017). Therefore, it is crucial to provide MHL and address the needs of all students, including COPMI.

Considerations for MHL programs included the following: ensuring students have an ability to self-reflect on the content, collaborating with students during implementation, resource availability, and including multimodal delivery. School staff suggested MHL programs be integrated into a whole school approach, providing resources and training to staff implementing the program, and involving families. Allied health professionals suggested strategies based on established therapeutic techniques, such as cognitive behavior therapy, ensuring programs are contextually appropriate and focusing on positive mental health. Including a guest with lived experience of mental illness was suggested; however, contact-based MHL interventions have been shown to be less effective than educational MHL interventions (Seedaket et al., 2020).

### Limitations and Future Directions

There are a few limitations of the current study. Firstly, the quantitative items in the survey were not tested for validity or reliability; therefore, the study findings may be limited due to the lack of pilot testing of items (O’Connor & Joffe, 2020). However, as the items were developed to provide descriptive data and qualitatively derived (Braun et al., 2020), findings help to explore the current perspectives and experiences of participants and allow some findings to be quantified. Secondly, social media was used to recruit participants for the current study. Results are limited to individuals with access to internet and who use social media. Researching using social media has many benefits, including the ability to reach a wide range of people with a fast and affordable approach (Grové, 2019). Specifically, recruiting participants for online questionnaires via social media provides a feasible and efficient tool (Fenner et al., 2012). Challenges to using social media to recruit participants are that participants may not be representative of the population, and this may introduce sample bias affecting the validity and reliability of results (Gelinas et al., 2017; Grové, 2019). Participants in this study were aware of the focus of the study and may have held biases towards the importance of including mental health education in schools. Whilst research using social media recruitment has its advantages, these limitations must be considered in light of the findings.

Lastly, the sample size was small and contained mostly female participants. As the study is qualitatively designed and informed, the aim of recruitment was not to achieve a statistically representative sample. Instead, the aim was to attain a saturation of themes of data collection until no new themes emerge (Saunders et al., 2018). This means that during the data collection phase, new data was gathered until it repeated what was expressed in previous responses. Although the sample was mostly female, the field of school staff and allied health professionals in Australia is predominantly female, with 71.6% of the education workforce and 77.9% of the health care and social assistance workforce being female (Australian Bureau of Statistics, 2020).

Implications for future research include the recruitment of participants through various means such as schools and community organizations, and the use of interviews to gain a richer insight into the perspectives of school staff and allied health professionals towards MHL and mental health in schools.

## Conclusion

Research targeting MHL and mental health education to reduce the prevalence of mental illness in youth is growing. For programs to be sustainable and feasible, it is essential to collaborate with key stakeholders involved in educating and supporting young people, such as teachers and school psychologists (McDaid et al., 2017). Though the key stakeholders in the current study (Australian school staff and allied health professionals) view mental health education as important in the school environment, there are barriers to limited resources and training for implementation. Based on the findings of this study, several suggestions are recommended. School staff require adequate training in youth mental health, including how to identify and support students at risk of developing mental illness, and increased awareness and implementation of evidence-based mental health programs in schools are needed. Participants in this study want schools to have clear policies and procedures for teaching and promoting mental health in the school environment with appropriate language and content provided. Allied health professionals, such as school psychologists, should be involved in supporting school staff to develop student MHL and implementing whole-school approaches to addressing MHL.

Mental illness stigma is still present in schools; however, MHL programs can address this by reducing negative attitudes towards mental health (Marinucci et al., 2021; Wei et al., 2013). The key stakeholders in this study viewed MHL content of mental health knowledge, seeking help, coping strategies, goal setting, and understanding the therapy process as important to teach young people in the school environment. Given the rising levels of psychological distress among youth (Brennan et al., 2021), preventative approaches such as MHL programs need to be implemented in school environments, and barriers to supporting youth mental health within schools must be addressed.

## Supplementary Information

ESM 1 Supplementary tables

## References

[CR1] American Psychological Association. (2022). *School psychology*. https://www.apa.org/ed/graduate/specialize/school

[CR2] Ashdown DM, Bernard ME (2012). Can explicit instruction in social and emotional learning skills benefit the social-emotional development, well-being, and academic achievement of young children?. Early Childhood Education Journal.

[CR3] Askell-Williams H, Cefai C (2014). Australian and Maltese teachers’ perspectives about their capabilities for mental health promotion in school settings. Teaching and Teacher Education.

[CR4] Australian Bureau of Statistics. (2020). *Gender indicators, Australia*. https://www.abs.gov.au/statistics/people/people-and-communities/gender-indicators-australia/latest-release

[CR5] Australian Curriculum, Assessment and Reporting Authority. (2021). *Australian curriculum*. https://www.australiancurriculum.edu.au/

[CR6] Australian Institute of Health Welfare. (2021). *Mental health services in Australia*. https://www.aihw.gov.au/reports/mental-health-services/mental-health-services-in-australia

[CR7] Australian Psychological Society (2016). *The framework for effective delivery of school psychology services: A practice guide for psychologists and school leaders*.

[CR8] Australian Psychological Society (2022). *Prevent, respond, adapt: Improving the mental health and wellbeing of all Australians*.

[CR9] Bale J, Grové C, Costello S (2020). Building a mental health literacy model and verbal scale for children: Results of a Delphi study. Children and Youth Services Review.

[CR10] Beames, J. R., Johnston, L., O’Dea, B., Torok, M., Boydell, K., Christensen, H., & Werner-Seidler, A. (2020). Addressing the mental health of school students: Perspectives of secondary school teachers and counselors [Article]. *International Journal of School and Educational Psychology.*10.1080/21683603.2020.1838367

[CR11] Berry Street. (2021). Berry street education model. https://www.berrystreet.org.au/learning-and-resources/berry-street-education-model

[CR12] Berzin SC, O'Brien KHM, Frey A, Kelly MS, Alvarez ME, Shaffer GL (2011). Meeting the social and behavioral health needs of students: Rethinking the relationship between teachers and school social workers. Journal of School Health.

[CR13] Beyond Blue. (2021). Be you. Australian Government. https://beyou.edu.au/

[CR14] Black Dog Institute. (2021). Education & services*:* Schools. https://www.blackdoginstitute.org.au/education-services/schools/

[CR15] Braun, V., Clarke, V., Boulton, E., Davey, L., & McEvoy, C. (2020). The online survey as a qualitative research tool. *International Journal of Social Research Methodology*, 1–14. 10.1080/13645579.2020.1805550

[CR16] Braun V, Clarke V (2006). Using thematic analysis in psychology [Internet]. Qualitative Research in Psychology.

[CR17] Braun V, Clarke V, Cooper H, Camic PM, Long DL, Panter AT, Rindskopf D, Sher KJ (2012). Thematic analysis. *APA handbook of research methods in psychology, Vol 2: Research designs: Quantitative, qualitative, neuropsychological, and biological* (pp. 57-71).

[CR18] Braun V, Clarke V (2014). What can “thematic analysis” offer health and wellbeing researchers?. International Journal of Qualitative Studies on Health and Well-being.

[CR19] Braun V, Clarke V (2021). *
Thematic analysis: A practical guide
*.

[CR20] Brennan N, Beames JR, Kos JR, Reily N, Connell C, Hall S, Christie K (2021). *
Psychological distress in young people in Australia: Fifth biennial youth mental health report: 2012-2020
*.

[CR21] Briesch AM, Sanetti LMH, Briesch JM (2010). Reducing the prevalence of anxiety in children and adolescents: An evaluation of the evidence base for the FRIENDS for Life program. School Mental Health: A Multidisciplinary Research and Practice Journal.

[CR22] Byrne A, Barber R, Lim CH (2021). Impact of the COVID-19 pandemic – A mental health service perspective. Progress in Neurology and Psychiatry.

[CR23] Cefai, C., Simões, C., & Caravita, S. (2021). A systemic, whole-school approach to mental health and wellbeing in schools in the EU: NESET report. *Publications Office of the European Union.*10.2766/50546

[CR24] Chandra A, Minkovitz CS (2007). Factors that influence mental health stigma among 8th grade adolescents. Journal of Youth and Adolescence.

[CR25] Chesterson J, Barker P (2009). Mental health promotion and prevention. Psychiatric and mental health nursing - The craft of caring.

[CR26] Cheung, K. K. C., & Tai, K. W. H. (2021). The use of intercoder reliability in qualitative interview data analysis in science education. *Research in Science & Technological Education*, 1–21. 10.1080/02635143.2021.1993179

[CR27] Ciarrochi, J., Atkins, P. W. B., Hayes, L. L., Sahdra, B. K., & Parker, P. (2016). Contextual positive psychology: Policy recommendations for implementing positive psychology into schools. *Frontiers in psychology, 7*(1561). 10.3389/fpsyg.2016.01561 10.3389/fpsyg.2016.01561 PMC5056194 27777564

[CR28] Collaborative for Academic, Social, and Emotional Learning. (2021). *What is SEL?* Collaborative for academic, social, and emotional learning (CASEL). https://casel.org/what-is-sel/

[CR29] Conley CS, Durlak JA, Bährer-Kohler S, Carod-Artal FJ (2017). Universal mental health promotion and prevention programs for students. *Global mental health: Prevention and promotion*.

[CR30] Corcoran T, Finney D (2015). Between education and psychology: School staff perspectives [Article]. Emotional and Behavioural Difficulties.

[CR31] Cowie H, Boardman C, Dawkins J, Jennifer D (2004). *Emotional health and well-being: A practical guide for schools*.

[CR32] Cross D, Shaw T, Epstein M, Pearce N, Barnes A, Burns S, Runions K (2018). Impact of the friendly schools whole-school intervention on transition to secondary school and adolescent bullying behaviour. European Journal of Education.

[CR33] Department of Education. (2021a). Positive behaviour for learning. New South Wales Government. https://education.nsw.gov.au/student-wellbeing/attendance-behaviour-and-engagement/positive-behaviour-for-learning/what-is-positive-behaviour-for-learning-pbl

[CR34] Department of Education. (2021b). School-wide positive behaviour support framework. Victoria Government. https://www2.education.vic.gov.au/pal/behaviour-students/guidance/5-school-wide-positive-behaviour-support-swpbs-framework

[CR35] Dimitropoulos, G., Cullen, E., Cullen, O., Pawluk, C., McLuckie, A., Patten, S., . . . Arnold, P. D. (2021). “Teachers often see the red flags first”: Perceptions of school staff regarding their roles in supporting students with mental health concerns. *School Mental Health*10.1007/s12310-021-09475-1

[CR36] Dix K, Kashfee SA, Carslake T, Sneidze-Gregory S, O’Grady E, Trevitt J (2020). A systematic review of intervention research examining effective student wellbeing in schools and their academic outcomes [Internet].

[CR37] Educator Impact. (2018). *The wellbeing and culture platform for schools*. https://www.educatorimpact.com/

[CR38] Ekornes S (2015). Teacher perspectives on their role and the challenges of inter-professional collaboration in mental health promotion [Article]. School Mental Health.

[CR39] Ekornes S (2017). Teacher stress related to student mental health promotion: The match between perceived demands and competence to help students with mental health problems. Scandinavian Journal of Educational Research.

[CR40] Feinstein NR, Fielding K, Udvari-Solner A, Joshi SV (2009). The supporting alliance in child and adolescent treatment: Enhancing collaboration among therapists, parents, and teachers [Article]. American Journal of Psychotherapy.

[CR41] Fenner Y, Garland SM, Moore EE, Jayasinghe Y, Fletcher A, Tabrizi SN, Wark JD (2012). Web-based recruiting for health research using a social networking site: an exploratory study. J Med Internet Res.

[CR42] Frydenberg E, Lewis R, Bugalski K, Cotta A, McCarthy C, Luscombe-Smith N, Poole C (2004). Prevention is better than cure: Coping skills training for adolescents at school. Educational Psychology in Practice.

[CR43] Frydenberg E, Muller D, Ivens C (2006). The experience of loss: Coping and the seasons for growth program. The Australian Educational and Developmental Psychologist.

[CR44] Gelinas L, Pierce R, Winkler S, Cohen IG, Lynch HF, Bierer BE (2017). Using social media as a research recruitment tool: ethical issues and recommendations. The American Journal of Bioethics.

[CR45] Graham A, Phelps R, Maddison C, Fitzgerald R (2011). Supporting children’s mental health in schools: Teacher views [Article]. Teachers and Teaching: Theory and Practice.

[CR46] Grové, C. (2019). Using social networking sites in research: an emerging approach to engaging with young people who have a parent with a mental illness and/or Substance abuse disorder [Brief Research Report]. *Frontiers in Psychiatry, 10*. 10.3389/fpsyt.2019.00281 10.3389/fpsyt.2019.00281 PMC6506785 31118908

[CR47] Grové C, Laletas S (2020). Promoting student wellbeing and mental health through social and emotional learning. Inclusive Education for the 21st Century: Theory, Policy and Practice.

[CR48] Grové C, Reupert A, Maybery D (2016). The perspectives of young people of parents with a mental illness regarding preferred interventions and supports. Journal of Child and Family Studies.

[CR49] Grové C, Riebschleger J, Bosch A, Cavanaugh D, van der Ende PC (2017). Expert views of children’s knowledge needs regarding parental mental illness. Children and Youth Services Review.

[CR50] Hagermoser Sanetti LM, Collier-Meek MA (2019). Increasing implementation science literacy to address the research-to-practice gap in school psychology. Journal of School Psychology.

[CR51] Hall S, Fildes J, Perrens B, Plummer J, Carlisle E, Cockayne N, Werner-Seidler A (2019). *Can we talk? Seven year youth mental health report - 2012-2018*.

[CR52] Hart LM, Mason RJ, Kelly CM, Cvetkovski S, Jorm AF (2016). ‘Teen mental health first aid’: A description of the program and an initial evaluation. International Journal of Mental Health Systems.

[CR53] headspace. (2021). *SAFEMinds*. https://safeminds.org.au/

[CR54] Hoare E, Thorp A, Bartholomeusz-Raymond N, McCoy A, Berk M (2020). Opportunities in the Australian national education initiative for promoting mental health in schools. The Lancet Child & Adolescent Health.

[CR55] Hudson J, Ingram V (2017). *Stigma-reduction and help-seeking in Australian classrooms: A research report on the batyr@school program*.

[CR56] Humphrey N, Barlow A, Wigelsworth M, Lendrum A, Pert K, Joyce C, Turner A (2016). A cluster randomized controlled trial of the promoting alternative thinking strategies (PATHS) curriculum. Journal of School Psychology.

[CR57] IBM. (2021). *SPSS statistics*. In [Computer software]. IBM. https://www.ibm.com/au-en/products/spss-statistics

[CR58] Interventions Plus. (2017). *RAGE*. https://www.interventionsplus.com.au/rage-re-navigating-anger-and-guilty-emotions/

[CR59] Independent Schools Victoria. (2021). *Cognizance*. https://is.vic.edu.au/programs/cognizance/

[CR60] Islam MI, Khanam R, Kabir E (2020). The use of mental health services by Australian adolescents with mental disorders and suicidality: Findings from a nationwide cross-sectional survey. PLoS ONE.

[CR61] Jorm AF (2012). Mental health literacy: Empowering the community to take action for better mental health. American Psychologist.

[CR62] Jorm AF, Okan O, Bauer U, Levin-Zamir D, Pinheiro P, Sorensen K (2019). The concept of mental health literacy. *
International handbook of health literacy: Research, practice and policy across the lifespan
*.

[CR63] Jorm AF, Kitchener BA (2020). Increases in youth mental health services in Australia: Have they had an impact on youth population mental health?. Australian & New Zealand Journal of Psychiatry.

[CR64] Jorm, A. F. (2020). We need to move from ‘mental health literacy’ to ‘mental health action’. *Mental Health & Prevention, 18*. 10.1016/j.mhp.2020.200179

[CR65] Jorm AF, Korten AE, Jacomb PA, Christensen H, Rodgers B, Pollitt P (1997). “Mental health literacy”: A survey of the public’s ability to recognise mental disorders and their beliefs about the effectiveness of treatment. Medical Journal of Australia.

[CR66] Kearney S, Gleeson C, Leung L, Ollis D, Joyce A (2016). *Respectful relationships education in schools: The beginnings of change: Final evaluation report*.

[CR67] Kretlow AG, Blatz SL (2011). The A B Cs of evidence-based practice for teachers. Teaching exceptional children.

[CR68] Kutcher S, Wei Y, Costa S, GusmГЈo R, Skokauskas N, Sourander A (2016). Enhancing mental health literacy in young people. European child & adolescent psychiatry.

[CR69] Laurens KR, Graham LJ, Dix KL, Harris F, Tzoumakis S, Williams KE, Green MJ (2021). School-based mental health promotion and early intervention programs in New South Wales.

[CR70] Lawrence D, Johnson S, Hafekost J, Boterhoven de Haan K, Sawyer M, Ainley J, Zubrick SR (2015). The mental health of children and adolescents: Report on the second Australian child and adolescent survey of mental health and wellbeing.

[CR71] Lindow JC, Hughes JL, South C, Minhajuddin A, Gutierrez L, Bannister E, Byerly MJ (2020). The youth aware of mental health intervention: Impact on help seeking, mental health knowledge, and stigma in U.S. adolescents. Journal of Adolescent Health.

[CR72] Mælan EN, Tjomsland HE, Baklien B, Thurston M (2020). Helping teachers support pupils with mental health problems through inter-professional collaboration: A qualitative study of teachers and school principals [Article]. Scandinavian Journal of Educational Research.

[CR73] Manicavasagar V, Horswood D, Burckhardt R, Lum A, Hadzi-Pavlovic D, Parker G (2014). Feasibility and effectiveness of a web-based positive psychology program for youth mental health: Randomized controlled trial. Journal of Medical Internet Research.

[CR74] Marinucci A, Grové C, Allen K-A, Riebschleger J (2021). Evaluation of a youth mental health literacy and action program: Protocol for a cluster controlled trial. Mental Health & Prevention.

[CR75] Marinucci, A., Grové, C., & Allen, K.-A. (2022a). A scoping review and analysis of mental health literacy interventions for children and youth. *School Psychology Review*, 1–15. 10.1080/2372966X.2021.2018918

[CR76] Marinucci, A., Grové, C., & Rozendorn, G. (2022b). “It’s something that we all need to know”: Australian youth perspectives of mental health literacy and action in schools [Original Research]. *Frontiers in Education, 7*. 10.3389/feduc.2022.829578

[CR77] Maybery DJ, Reupert AE, Patrick K, Goodyear M, Crase L (2009). Prevalence of parental mental illness in Australian families. Psychiatric Bulletin.

[CR78] Mazzer KR, Rickwood DJ (2015). Teachers role breadth and perceived efficacy in supporting student mental health [Article]. Advances in School Mental Health Promotion.

[CR79] McAllister M, Withyman C, Knight BA (2018). Facilitation as a vital skill in mental health promotion: Findings from a mixed methods evaluation. Journal of Mental Health Training, Education and Practice.

[CR80] McDaid, D., Hewlett, E., & Park, A. (2017). Understanding effective approaches to promoting mental health and preventing mental illness. *Organisation for Economic Cooperation and Development (OECD), 97*. 10.1787/bc364fb2-en

[CR81] McGorry P, Bates T, Birchwood M (2013). Designing youth mental health services for the 21st century: Examples from Australia, Ireland and the UK. British Journal of Psychiatry.

[CR82] Moon J, Williford A, Mendenhall A (2017). Educators' perceptions of youth mental health: Implications for training and the promotion of mental health services in schools. Children and Youth Services Review.

[CR83] Moses T (2010). Being treated differently: Stigma experiences with family, peers, and school staff among adolescents with mental health disorders. Social Science & Medicine.

[CR84] Myles-Pallister, J. D., Hassan, S., Rooney, R. M., & Kane, R. T. (2014). The efficacy of the enhanced Aussie Optimism Positive Thinking Skills Program in improving social and emotional learning in middle childhood. *Frontiers in psychology, 5*(909). 10.3389/fpsyg.2014.00909 10.3389/fpsyg.2014.00909 PMC4133646 25177310

[CR85] NAPCAN. (2021). *Love bites*. https://www.napcan.org.au/Programs/love-bites/

[CR86] National Association of School Psychologists (2021). *Who are school psychologists?*.

[CR87] National Mental Health Commission (2021). *The national children’s mental health and wellbeing strategy*.

[CR88] National Mental Health Commission (2022). *Vision 2030: Blueprint for mental health and suicide prevention*.

[CR89] Noble T, McGrath H, Wosnitza M, Peixoto F, Beltman S, Mansfield CF (2018). Making it real and making it last! Sustainability of teacher implementation of a whole-school resilience programme. *Resilience in Education: Concepts, Contexts and Connections*.

[CR90] Nowell SW, Watson LR, Boyd B, Klinger LG (2019). Efficacy study of a social communication and self-regulation intervention for school-age children with autism spectrum disorder: A randomized controlled trial. Language, Speech, & Hearing Services in Schools.

[CR91] O’Connor C, Joffe H (2020). Intercoder reliability in qualitative research: Debates and practical guidelines. International Journal of Qualitative Methods.

[CR92] O’Reilly M, Adams S, Whiteman N, Hughes J, Reilly P, Dogra N (2018). Whose responsibility is adolescent’s mental health in the UK? Perspectives of key stakeholders. School Mental Health.

[CR93] O'Connor CA, Dyson J, Cowdell F, Watson R (2018). Do universal school-based mental health promotion programmes improve the mental health and emotional wellbeing of young people? A literature review. Journal of clinical nursing.

[CR94] Ojio Y, Foo JC, Usami S, Fuyama T, Ashikawa M, Ohnuma K, Sasaki T (2019). Effects of a school teacher-led 45-minute educational program for mental health literacy in pre-teens. Early Intervention in Psychiatry.

[CR95] Painter K, Phelan JC, DuPont-Reyes MJ, Barkin KF, Villatoro AP, Link BG (2017). Evaluation of antistigma interventions with sixth-grade students: A school-based field experiment. Psychiatric Services.

[CR96] Patalay P, Annis J, Sharpe H, Newman R, Main D, Ragunathan T, Clarke K (2017). A pre-post evaluation of OpenMinds: A sustainable, peer-led mental health literacy programme in universities and secondary schools. Prevention Science.

[CR97] Perry Y, Petrie K, Buckley H, Cavanagh L, Clarke D, Winslade M, Christensen H (2014). Effects of a classroom-based educational resource on adolescent mental health literacy: A cluster randomised controlled trial. Journal of Adolescence.

[CR98] Plano Clark VL (2019). Meaningful integration within mixed methods studies: Identifying why, what, when, and how. Contemporary Educational Psychology.

[CR99] Powell TM, Bui T (2016). Supporting social and emotional skills after a disaster: Findings from a mixed methods study. School Mental Health.

[CR100] Prasun MA (2013). Evidence-based practice. Heart & Lung.

[CR101] Psychology Board of Australia (2020). *General registration*.

[CR102] Qualtrics. (2021). *Qualtrics*. In (Version July 2021) https://www.qualtrics.com

[CR103] Radez, J., Reardon, T., Creswell, C., Lawrence, P. J., Evdoka-Burton, G., & Waite, P. (2020). Why do children and adolescents (not) seek and access professional help for their mental health problems? A systematic review of quantitative and qualitative studies. *European child & adolescent psychiatry.*10.1007/s00787-019-01469-4 10.1007/s00787-019-01469-4 PMC7932953 31965309

[CR104] Radez, J., Reardon, T., Creswell, C., Orchard, F., & Waite, P. (2021). Adolescents’ perceived barriers and facilitators to seeking and accessing professional help for anxiety and depressive disorders: A qualitative interview study. *European child & adolescent psychiatry.*10.1007/s00787-020-01707-0 10.1007/s00787-020-01707-0 PMC9209355 33502596

[CR105] RealWell (2021). *Rumble’s quest*.

[CR106] Reinke WM, Stormont M, Herman KC, Puri R, Goel N (2011). Supporting children's mental health in schools: Teacher perceptions of needs, roles, and barriers. School Psychology Quarterly.

[CR107] Reupert A, Schaffer GE, Von Hagen A, Allen K-A, Berger E, Büttner G (2022). The practices of psychologists working in schools during COVID-19: A multi-country investigation. School Psychology.

[CR108] Rose, J. (2004). Protective Behaviours: safety, confidence and self-esteem. *Journal of Public Mental Health, 3*(1). 10.1108/17465729200400004

[CR109] Russet, F., Humbertclaude, V., Davidovic Vrljicak, N., Dieleman, G. C., Dodig-Ćurković, K., Franic, T., . . . Purper-Ouakil, D. (2022). Are psychiatrists trained to address the mental health needs of young people transitioning from child to adult services? Insights from a European Survey [Original Research].. Frontiers in Psychiatry, 12 10.3389/fpsyt.2021.768206 10.3389/fpsyt.2021.768206 PMC8864158 35222101

[CR110] Samnøy, S., Thurston, M., Wold, B., Jenssen, E. S., & Tjomsland, H. E. (2020). Schooling as a contribution or threat to wellbeing? A study of Norwegian teachers’ perceptions of their role in fostering student wellbeing [Article]. *Pastoral Care in Education.*10.1080/02643944.2020.1855673

[CR111] Santor DA, Bagnell AL (2012). Maximizing the uptake and sustainability of school-based mental health programs: Commercializing knowledge. Child and adolescent psychiatric clinics of North America.

[CR112] Saunders B, Sim J, Kingstone T, Baker S, Waterfield J, Bartlam B, Jinks C (2018). Saturation in qualitative research: Exploring its conceptualization and operationalization. Quality & Quantity.

[CR113] Schonert-Reichl KA, Oberle E, Lawlor MS, Abbott D, Thomson K, Oberlander TF, Diamond A (2015). Enhancing cognitive and social-emotional development through a simple-to-administer mindfulness-based school program for elementary school children: A randomized controlled trial. Developmental psychology.

[CR114] Seedaket S, Turnbull N, Phajan T, Wanchai A (2020). Improving mental health literacy in adolescents: Systematic review of supporting intervention studies [Internet]. Tropical Medicine & International Health.

[CR115] Serafini G, Parmigiani B, Amerio A, Aguglia A, Sher L, Amore M (2020). The psychological impact of COVID-19 on the mental health in the general population. QJM: An International Journal of Medicine.

[CR116] Singh S, Roy D, Sinha K, Parveen S, Sharma G, Joshi G (2020). Impact of COVID-19 and lockdown on mental health of children and adolescents: A narrative review with recommendations. Psychiatry Research.

[CR117] Smiling Mind (2021). *Smiling mind*.

[CR118] Soutter M (2018). *The role of the Leader in me in the social and emotional learning and youth voice development of elementary students* (Publication Number 10751062) [Doctoral Dissertation, Boston University].

[CR119] The Man Cave. (2021). *Insights & impact report*. https://themancave.life/our-results/

[CR120] The Resilience Project. (2021). *University of Melbourne evaluation*. https://theresilienceproject.com.au/university-of-melbourne-research-findings/

[CR121] Townsend L, Musci R, Stuart E, Ruble A, Beaudry MB, Schweizer B, Swartz K (2017). The association of school climate, depression literacy, and mental health stigma among high school students. Journal of School Health.

[CR122] United Nations. (1989). Convention of the rights of the child. *In.*

[CR123] Weare K, Bahrer-Kohler S, Carod-Artal FJ (2017). Promoting social and emotional wellbeing and responding to mental health problems in schools. *Global mental health: Prevention and promotion*.

[CR124] Wei Y, Hayden JA, Kutcher S, Zygmunt A, McGrath P (2013). The effectiveness of school mental health literacy programs to address knowledge, attitudes and help seeking among youth. Early Intervention in Psychiatry.

[CR125] Whitley J, Smith JD, Vaillancourt T (2013). Promoting mental health literacy among educators: Critical in school-based prevention and intervention. Canadian Journal of School Psychology.

[CR126] Wheel of Well-Being. (2013). *Wheel of well-being*. https://www.wheelofwellbeing.org/

[CR127] World Health Organization (2002). *Prevention and promotion in mental health*.

[CR128] World Health Organization (2005). *Promoting mental health: Concepts, emerging evidence, practice*.

[CR129] World Health Organization (2018). *Mental health: Strengthening our response*.

[CR130] Wyn J, Cahill H, Holdsworth R, Rowling L, Carson S (2000). MindMatters, a whole-school approach promoting mental health and wellbeing. Australian & New Zealand Journal of Psychiatry.

[CR131] Ykema F, Hartman D, Imms W (2006). Bringing it together. Includes 22 case studies of Rock & Water in practice in various settings.

[CR132] yourtown. (2021). *Our services*. https://www.yourtown.com.au/our-services?filter_audience=learning

