# Supplementary material for: Australian School Staff and Allied Health Professional Perspectives of Mental Health Literacy in Schools: a Mixed Methods Study
Source: Educ Psychol Rev. 2023 Jan 21;35(1):3. doi: 10.1007/s10648-023-09725-5 (PMC9869299; doi:10.1007/s10648-023-09725-5)
Supplement: Supplementary file 1 — Supplementary tables [file 10648_2023_9725_MOESM1_ESM.docx]

Supplementary Information

| **Table S.1**  *Survey items* | | |
| --- | --- | --- |
| Quantitative Items | | |
| Item | Scale |  |
| Mental health is stigmatised in the school environment | Strongly disagree – Strongly agree |  |
| It is difficult to talk about mental health at school with students |  |  |
| Parents do not want school staff to discuss mental health at school |  |  |
| There are barriers to teachers implementing mental health promotion in schools |  |  |
| My professional training addressed mental health education for students |  |  |
| Schools place high importance on the mental health of students |  |  |
| I feel competent delivering mental health education to students |  |  |
| It is part of a teacher's role to deliver mental health education |  |  |
| My school has a clear policy on the type of information we teach about mental health |  |  |
| School-based mental health initiatives have the capacity to strengthen student’s academic performance |  |  |
| School-based mental health initiatives have the capacity to strengthen student’s mental health and wellbeing |  |  |
| School-based mental health initiatives have the capacity to strengthen student’s resilience |  |  |
| School-based mental health initiatives have the capacity to strengthen student’s help-seeking behaviour |  |  |
| School-based mental health initiatives have the capacity to strengthen student’s adaptive coping behaviours |  |  |
| School-based mental health initiatives have the capacity to strengthen student’s responding to others |  |  |
| Schools need to take a preventative approach to mental health | Extremely important – Not at all important |  |
| Students need to be aware of where to seek help for mental health problems |  |  |
| Mental health education should be a part of the curriculum |  |  |
| Teachers need adequate training to manage child and adolescent mental health and wellbeing |  |  |
| Teachers need to understand mental health problems that students may experience |  |  |
| In your experience/professional opinion, how useful are the following types of delivery for mental health information? | Extremely useful – Not at all useful |  |
| Posters |  |  |
| Brochures |  |  |
| Lectures/talks from external providers |  |  |
| Class discussions |  |  |
| Informal discussions with students |  |  |
| Workshops |  |  |
| Directing students to websites |  |  |
| Formal classes addressing mental health education |  |  |
| Who of the following school staff should be involved in mental health education? | Multiple choice |  |
| Classroom teacher |  |  |
| Wellbeing coordinator |  |  |
| School counsellor/Mental health practitioner/School psychologist |  |  |
| Vice principal |  |  |
| Health teacher |  |  |
| None of the above |  |  |
| If mental health literacy were incorporated into the school curriculum, what would you need to deliver this?/what do you think school staff would need to deliver this? | Multiple choice |  |
| Professional development training in mental health literacy |  |  |
| Support from administrators |  |  |
| Extra planning time |  |  |
| Resources (e.g. teaching materials) |  |  |
| Inclusive, safe and supportive school community |  |  |
| Other (open response) |  |  |
| Qualitative Items | |  |
| What is covered in health education associated with mental health? Is it helpful? Why/Why not?  What school-based mental health programs, if any, have been implemented in your school?  The structure of the YES program with objectives for each session are as follows:  Week 1: Introduction to YES   - Understand what to expect in the YES program - Describe stress (physical sensations, triggers) - Develop group values and expectations during the program   Week 2: Introduction to Coping   - Describe coping behaviours - Create a coping behaviour plan   Week 3: Mental Illness & Recovery   - Understand definition and prevalence of mental illness - Understand heritability and recovery process for mental illness   What, if anything, do you like about Week 1, 2 and 3 of the YES program?  What, if anything, do you not like about Week 1, 2 and 3 of the YES program?  Is there anything you would change about Week 1, 2 and 3 of the YES program? Please explain.  Week 4: Depression & Anxiety   - Understand emotions and behaviour associated with depression - Learn some strategies to manage depressive and/or anxious symptoms   Week 5: Coping & Resilience   - Develop individual coping strategies - Understand healthy behaviours to promote resilience and coping   What, if anything, do you like about Week 4 and 5 of the YES program?  What, if anything, do you not like about Week 4 and 5 of the YES program?  Is there anything you would change about Week 4 and 5 of the YES program? Please explain.  Week 6: Help Seeking & Support   - Understand where to seek help (formal and informal) - Understand how to seek help (e.g. helplines, community, professional) - Understand how to help others who may experience mental illness - Understand process of therapy and what to expect   Week 7: Stigma   - Understand stigmatised views of people with mental illness - Understand how stigmatised people are treated   What, if anything, do you like about Week 6 and 7 of the YES program?  What, if anything, do you not like about Week 6 and 7 of the YES program?  Is there anything you would change about Week 6 and 7 of the YES program? Please explain.  Week 8: Families   - Understand how mental illness and stigma can affect families   Week 9: Values & Goals   - Exploring values - Goal setting directed by values - Demonstrate understanding of mental health literacy   Week 10: YES graduation   - Describe what learnt during YES - Reflect on coping skills - Describe how will use YES learning in future   What, if anything, do you like about Week 8, 9 and 10 of the YES program?  What, if anything, do you not like about Week 8, 9 and 10 of the YES program?  Is there anything you would change about Week 8, 9 and 10 of the YES program? Please explain.  Are there any areas of mental health education that are missing from the YES program? | |  |

| **Table S.2**  *Educators (n = 52), allied health professionals (n = 36), and overall sample (n = 88) mean (M) and standard deviation (SD) for items rated strongly disagree (1) to strongly agree (5)* | | | | |
| --- | --- | --- | --- | --- |
| Item |  | Educators | Allied health professionals | Overall Sample |
| Mental health initiatives can strengthen mental health | *M* | 4.38 | 4.69 | 4.51 |
|  | *SD* | 1.11 | .86 | 1.02 |
| Mental health initiatives can strengthen help-seeking | *M* | 4.27 | 4.50 | 4.36 |
|  | *SD* | 1.11 | .85 | 1.01 |
| Mental health initiatives can strengthen responding to others | *M* | 4.23 | 4.47 | 4.33 |
|  | *SD* | 1.11 | .77 | .99 |
| Mental health initiatives can strengthen resilience | *M* | 4.23 | 4.47 | 4.33 |
|  | *SD* | 1.22 | .88 | 1.09 |
| Mental health initiatives can strengthen adaptive coping | *M* | 4.17 | 4.47 | 4.30 |
|  | *SD* | 1.15 | .85 | 1.04 |
| Mental health initiatives can strengthen academic performance* | *M* | 3.87 | 4.53 | 4.14 |
|  | *SD* | 1.37 | .77 | 1.21 |
| There are barriers to teachers implementing mental health promotion | *M* | 3.79 | 4.03 | 3.89 |
|  | *SD* | .94 | .77 | .88 |
| A teacher’s role is to deliver mental health education | *M* | 3.56 | 3.92 | 3.70 |
|  | *SD* | 1.18 | .91 | 1.08 |
| I feel competent to deliver mental health education* | *M* | 3.27 | 4.25 | 3.67 |
|  | *SD* | 1.09 | .77 | 1.08 |
| Schools place high importance on mental health | *M* | 3.25 | 3.72 | 3.44 |
|  | *SD* | 1.31 | 1.06 | 1.23 |
| Mental health is stigmatized in schools | *M* | 3.21 | 3.39 | 3.28 |
|  | *SD* | 1.21 | 1.15 | 1.18 |
| It is difficult to talk about mental health with students | *M* | 2.85 | 2.69 | 2.78 |
|  | *SD* | 1.32 | 1.31 | 1.31 |
| Parents are reluctant to mental health education | *M* | 2.88 | 2.53 | 2.74 |
|  | *SD* | 1.02 | 1.18 | 1.10 |
| My school has a clear policy on mental health education | *M* | 2.58 | 2.81 | 2.67 |
|  | *SD* | 1.26 | 1.09 | 1.19 |
| My professional training addressed mental health education* | *M* | 1.96 | 3.58 | 2.63 |
|  | *SD* | 1.07 | 1.23 | 1.38 |
| *Allied health professionals and educators group means are significantly different.  *Note*: 1 = strongly disagree, 2 = somewhat disagree, 3 = neither agree nor disagree, 4 = somewhat agree, 5 = strongly agree | | | | |

| **Table S.3**  *Educators (n = 52), allied health professionals (n = 36), and overall sample (n = 88) mean (M) and standard deviation (SD) for items rated extremely important (1) to not at all important (5)* | | | | |
| --- | --- | --- | --- | --- |
| Item |  | Educators | Allied health professionals | Overall Sample |
| Mental health education should be part of the curriculum | *M* | 1.85 | 1.58 | 1.74 |
|  | *SD* | 1.04 | 1.05 | .98 |
| Teachers need adequate training | *M* | 1.52 | 1.64 | 1.57 |
|  | *SD* | .98 | 1.07 | 1.02 |
| Schools need to take a preventative approach | *M* | 1.58 | 1.44 | 1.52 |
|  | *SD* | .87 | 1.00 | .92 |
| Teachers need to understand mental health problems | *M* | 1.44 | 1.53 | 1.48 |
|  | *SD* | .87 | .97 | .91 |
| Students need to know where to seek help | *M* | 1.33 | 1.31 | 1.32 |
|  | *SD* | .73 | .95 | .82 |
| *Note*: 1 = extremely important, 2 = very important, 3 = moderately important, 4 = slightly important, 5 = not at all important | | | | |

| **Table S.4**  *Educators (n = 52), allied health professionals (n = 36), and overall sample (n = 88) mean (M) and standard deviation (SD) for items rated extremely useful (1) to not at all useful (5)* | | | | |
| --- | --- | --- | --- | --- |
| Item |  | Educators | Allied health professionals | Overall Sample |
| Brochures | *M* | 3.37 | 3.67 | 3.49 |
|  | *SD* | .89 | .99 | .94 |
| Posters | *M* | 3.33 | 3.17 | 3.26 |
|  | *SD* | .81 | .88 | .84 |
| Directing to websites | *M* | 3.35 | 3.11 | 3.25 |
|  | *SD* | .81 | .79 | .81 |
| Lectures from external providers | *M* | 2.31 | 2.33 | 2.32 |
|  | *SD* | 1.00 | .83 | .93 |
| Formal classes | *M* | 2.29 | 2.14 | 2.23 |
|  | *SD* | 1.11 | .96 | 1.05 |
| Workshops | *M* | 2.02 | 1.89 | 1.97 |
|  | *SD* | .96 | .75 | .88 |
| Class discussions | *M* | 1.90 | 1.78 | 1.85 |
|  | *SD* | .89 | .80 | .85 |
| Informal discussions | *M* | 1.75 | 1.61 | 1.69 |
|  | *SD* | .99 | .73 | .89 |
| *Note*: 1 = extremely useful, 2 = very useful, 3 = moderately useful, 4 = slightly useful, 5 = not at all useful | | | | |
